# High prevalence of *TP53* loss and whole-genome doubling in early-onset colorectal cancer

**DOI:** 10.1038/s12276-021-00583-1

**Published:** 2021-03-22

**Authors:** Jeong Eun Kim, Jaeyong Choi, Chang-Ohk Sung, Yong Sang Hong, Sun Young Kim, Hyunjung Lee, Tae Won Kim, Jong-Il Kim

**Affiliations:** 1 Department of Oncology, Asan Medical Center, University of Ulsan College of Medicine, Seoul, Korea; 2 Department of Biomedical Sciences, Seoul National University College of Medicine, Seoul, Korea; 3 Department of Pathology, Asan Medical Center, University of Ulsan College of Medicine, Seoul, Korea; 4 Asan Center for Cancer Genome Discovery, Asan Medical Center, University of Ulsan College of Medicine, Seoul, Korea; 5 Genomic Medicine Institute, Medical Research Center, Seoul National University, Seoul, Korea; 6 Cancer Research Institute, Seoul National University College of Medicine, Seoul, Korea

**Keywords:** Medical genomics, Colon cancer

## Abstract

The global incidence of early-onset colorectal cancer (EO-CRC) is rapidly rising. However, the reason for this rise in incidence as well as the genomic characteristics of EO-CRC remain largely unknown. We performed whole-exome sequencing in 47 cases of EO-CRC and targeted deep sequencing in 833 cases of CRC. Mutational profiles of EO-CRC were compared with previously published large-scale studies. EO-CRC and The Cancer Genome Atlas (TCGA) data were further investigated according to copy number profiles and mutation timing. We classified colorectal cancer into three subgroups: the hypermutated group consisted of mutations in *POLE* and mismatch repair genes; the whole-genome doubling group had early functional loss of *TP53* that led to whole-genome doubling and focal oncogene amplification; the genome-stable group had mutations in *APC* and *KRAS*, similar to conventional colon cancer. Among non-hypermutated samples, whole-genome doubling was more prevalent in early-onset than in late-onset disease (54% vs 38%, Fisher’s exact *P* = 0.04). More than half of non-hypermutated EO-CRC cases involved early *TP53* mutation and whole-genome doubling, which led to notable differences in mutation frequencies between age groups. Alternative carcinogenesis involving genomic instability via loss of *TP53* may be related to the rise in EO-CRC.

## Colorectal cancer: tumor genetic classification could help guide treatment

Researchers in South Korea have identified distinct types of genetic changes in colorectal cancer (CRC). Groups led by Tae Won Kim at the University of Ulsan, Seoul, and Jong-il Kim at Seoul National University looked for mutations in genes in tumors from 880 patients, including all active genes in 47 patients with early-onset colorectal cancer (EO-CRC). The cancers were classified into three subgroups. A “hypermutated” group had mutations in genes involved in DNA replication and repair. The entire genome was doubled in another group, which included the EO-CRC cases. In this group, *TP53* gene function, which is known to be crucial in suppressing tumor formation, was lost. A third group had mutations in two other identified genes. Identifying which subgroups patients belong to may help determine the best treatment option.

## Introduction

The incidence of colorectal cancer (CRC) is rising among individuals younger than 55 years old^1^, a trend observed in countries worldwide, including the US, China, Sweden, the UK, and Korea^2,3^. The American Cancer Society has lowered the recommended age for regular screening from 50 to 45 in people with average risks of CRC^4^, and increases in the screening rate via colonoscopy partly account for the global rise in the incidence of CRC. However, although the rate of colonoscopy rates decreased in the United States after 2009, the rate of early-onset colorectal cancer (EO-CRC) increased during the same period^5^. Hereditary cancer syndrome, in which a person carries cancer-predisposing germline variants and develops cancer in early in life, may also account for the increase in the incidence of EO-CRC. Nevertheless, previous studies have shown that the proportion of hereditary CRC in all EO-CRC is merely 10–20%^6,7^, indicating that the majority of EO-CRC cases are sporadic.

Clinically, EO-CRC is distinct from traditional late-onset CRC (LO-CRC) in that EO-CRC tends to occur more often in males and African Americans and in the left colon and involves more aggressive histology and higher stages. Contrary to the extensive amount of research carried out on the hereditary and environmental aspects of EO-CRC, there are few studies about the genomic aspects of sporadic EO-CRC^8,9^. Moreover, studies that have been performed used targeted sequencing, which has limitations in providing the full genomic nature of the disease.

Here, we present the full genomic landscape of EO-CRC and compare it with that of LO-CRC via integrated analysis of clinical data, mutational status, and copy number profiles. We performed whole-exome sequencing (WES) in 47 cases of EO-CRC and targeted deep sequencing in 833 cases of CRC (99 EO-CRC, 734 LO-CRC). We also carried out a combined analysis using our data and previously published data from large-scale CRC studies.

## Materials and methods

### Patient selection

To investigate genomic differences in CRC between age groups, we defined EO-CRC as CRC diagnosed before the age of 40 years. Among all patients who were treated with CRC in Asan Medical Center, those diagnosed with CRC younger than 40 years of age between June 2016 and December 2017 were included in the analysis. This study was approved by the Institutional Review Board of Asan Medical Center (#2016–0673) and conducted in accordance with the Declaration of Helsinki and the Guidelines for Good Clinical Practice. WES using tumor tissues and blood samples was carried out after obtaining informed consent from each patient. One 41-year-old patient was included due to error in age calculation resulting from delayed confirmation of the date of diagnosis using outside pathologic reports. Information on predisposing pathogenic germline variants, as well as other incidental findings recommended by the ACMG found during this study, was delivered to each corresponding patient, with the participant’s prior consent taken into account. Recommendations were made by the clinician, and genetic counseling was arranged for patients who wished to obtain further information.

### DNA purification and sequencing

Tumor samples were histologically examined with hematoxylin and eosin staining to determine tumor cellularity (≥50%), and the tumor areas were marked under a microscope. Genomic DNA from the corresponding formalin-fixed paraffin-embedded (FFPE) tissues was extracted using QIAamp DNA FFPE Tissue Kit (Qiagen Inc., CA, USA) according to the manufacturer’s instructions. Genomic DNA was eluted with DNase-/RNase-free water and quantified using a NanoDrop spectrophotometer and the PicoGreen system (Invitrogen Life Technologies, CA, USA). SureSelect Human All Exon V6 Kit (Agilent Technologies, CA, USA) was used to capture exon regions, and a sequencing library was generated according to the manufacturer’s instructions. Library quality was verified with an Agilent Bioanalyzer (Agilent). Paired-end 101-bp reads were sequenced with the Illumina HiSeq 2500 platform (Illumina, CA, USA).

### Sequence data analysis

The Illumina adapter was trimmed with Trimmomatic (v0.36)^10^, and the reads were aligned to the 1000 Genomes Project phase 1 version of the human reference genome with BWA-mem (v0.7.15)^11^. Picard (v2.6.0) was utilized to remove duplicate PCR reads and coordinately sort the reads. Indel realignment and base quality recalibration were performed with GATK (v3.8.0)^12^.

Germline single-nucleotide polymorphisms (SNPs) and indels were called with HaplotypeCaller using the allele-specific model. Variant Quality Score Recalibration (VQSR) was applied to filter sequencing errors. Somatic mutations were called with MuTect2. Copy number profiles were produced with CNVkit (v0.9.3)^13^. Large structural variants were called Lumpy (v0.2.13)^14^ and genotyped with svtyper (v0.5.2)^15^. Small complex structural variants were called with Pindel (v0.2.5.b9)^16^. SNP and indel annotation was performed with ANNOVAR (2016Feb01)^17^ and Oncotator (16April05)^18^. Structural variant annotation was performed with SnpEff (4.3t)^19^. All mutations in frequently mutated genes and all germline pathogenic variants were manually reviewed with Integrated Genome Viewer (v2.3). The mutational context of the tumor samples was decomposed into 30 known COSMIC signatures using deconstructSigs (v1.8.0)^20^. The SNP transition/transversion ratio and heterozygous/homozygous ratio were calculated using RTGtools (v3.7.1)^21^. Cross-sample contamination was measured with the Plink (v1.9)^22^ identify by descent test. Calculations for purity, ploidy, and purity-adjusted copy number profiles were carried out with PureCN (v1.8.1)^23^.

### Mutation pathogenicity estimation

All germline variants and somatic mutations with pathogenic/likely pathogenic annotation in Clinvar^24^, Intervar^25^, or with a COSMIC (v83) database report count of over 100 were annotated as “Known Pathogenicity”. Afterward, mutations were filtered by (1) genes curated to be related to colon cancer, (2) population allele frequency lower than 0.01, (3) Clinvar and Intervar annotation not benign, (4) mutation causing loss of function, (5) variant allele frequency ≥0.03 in the current sample set, (6) genotype quality higher than 20, (7) mutated read count ≥3, and (8) total coverage in the region ≥10. For SNP mutations in *TP53*, we also used the combined phenotype score in the PHANTM database^26^ to assess pathogenicity.

We further evaluated whether a germline variant had an effect on carcinogenesis. Germline variants with evidence of positive selection by a copy-neutral loss of heterozygosity event or another compound somatic mutation causing functional loss of the gene were annotated as “Possible Pathogenicity”. Truncating germline variants without evidence of contribution to cancer development were annotated as “Variant of Unknown Significance”.

### TCGA mutation frequency

TCGA data were downloaded from GDC Data Portal^27^. We used MuTect2 results to reduce bias resulting from differences in the calling method. Mutation data were reannotated using our pipeline and filtered by the following criteria: (1) population frequency ≤0.01 in 1000 Genomes Project phase 3 and gnomAD (v2.0.2), (2) variant not annotated as benign or likely benign in Clinvar or Intervar classification, (3) at least one forward read and one reverse read (to remove strand bias error), (4) fraction of oxygenated guanine ≤0.8, and (5) variant allele frequency higher than 0.01 and <0.97 (to remove subclonal or germline variants). We further filtered mutations to meet at least one of the following criteria to be considered as oncogenic driver mutations: (1) variants annotated as pathogenic and/or likely pathogenic in Clinvar and/or Intervar, (2) variants reported 10 or more times in the COSMIC database, and (3) truncating mutations in tumor-suppressor genes. After filtering, we reviewed variants with extreme variant allele frequencies and removed unlikely driver variants. Variants remaining after filtration was used to calculate the mutation frequency and mutation timing.

### MSK mutation frequency

MSK clinical information and mutation data were downloaded from the article supplement^28^. Only primary CRC sample data were used. Because strand information was not provided, we applied the same filtering step used for the TCGA data, except for the strand bias and fraction of oxygenated guanine.

### Cancer panel sequencing

Sequencing of the tumor samples was performed as previously described^29^. In brief, after a review of the matched H&E slides by a pathologist, genomic DNA was isolated using the NEXprep FFPE Tissue kit (#NexK-9000; Geneslabs Gyeonggi-do, Korea) according to the manufacturer’s protocol. DNA was quantified using the QubitTM dsDNA HS Assay kit (Thermo Fisher Scientific, MA, USA).

Targeted NGS was performed using the MiSeq platform (Illumina) with OncoPanel AMC version 3 (OP_AMCv3), which was designed at Asan Medical Center through SureDesign (Agilent Technologies) using the GRCh37 reference version^30^.

### Copy number clustering

Calculations for purity, ploidy, and purity-adjusted copy number profiles were carried out with PureCN (v1.8.1)^23^. To perform copy number profile clustering, we summarized copy numbers to arm-level states by averaging all copy numbers for each arm. Clustergrammer^31^ was used with the Euclidean distance and average linkage. Arm-level copy number data were only used for clustering and visualization. To determine the ploidy cutoff for whole-genome doubling (WGD) events, we used 2420 samples^32^ calculated with ABSOLUTE and generated an accuracy plot for each ploidy cutoff. Although 2.37 had the highest accuracy of 0.9785, we conservatively selected the cutoff by specificity over 99% and chose 2.5, which had a comparable accuracy of 0.9727.

### TCGA copy number profile

A GDC pipeline-processed TCGA bam file was obtained from Google Cloud Platform. As different exome capture kits may result in biased copy number calculation, we used the samples captured with the most commonly used kit, namely, SeqCap EZ HGSC VCRome Kit (Roche, Basel, Switzerland). Coverage data were calculated with Picard CollectHsMetrics. Germline SNPs in tumor and normal tissues were called with HaplotypeCaller. PureCN was used to determine purity, ploidy, and variant clonality. Among the 631 TCGA samples uploaded in GCS, 198 were captured with different kits, 63 did not have matched normal or tumor data, 33 had a prior cancer diagnosis, 15 had PureCN calculation failures, 2 had corrupted bam files, 2 were not colorectal carcinomas, and 1 involved neoadjuvant therapy. The final counts of 68 hypermutated and 250 non-hypermutated samples were analyzed.

### Mutation timing estimation

PureCN estimates the allele count of the mutation, the total allele counts of the mutated region, and the fraction of tumor cells carrying the mutation. We removed all mutations with cellular fractions below 50%. Each somatic mutation was assigned early, late, or unknown according to the following criteria: (1) if the region had a copy number state of higher than 2 and the mutation was present in >1 allele, we assumed that the mutation had occurred before copy number gain and classified the timing as “early”; (2) if the region had a copy number state of higher than 2 and the mutation was present in only 1 allele, we classified the timing as “late”; (3) if the region had exactly two copy numbers and both alleles had the mutation, we also classified the timing as “early”, as a mutation appearing before an loss of heterozygosity (LoH) event is more plausible than the same mutation arising from two independent events; (4) if the region had exactly two copy numbers and the mutation was present in only one allele while the region showed LoH, we classified the timing as “late”; and (5) all other instances in which the region had two copy numbers with one mutation present with no LoH event or the mutation was located in regions with less than two copy numbers were classified as “unknown”. The homozygous loss was classified if a mutation was present in all alleles or if the absolute mutated allele count was 7 or higher.

### Mismatch repair deficiency analysis

Mismatch repair status was determined using PCR or immunohistochemistry (IHC). For the detection of microsatellite instability (MSI) using PCR, 2~5 sections of FFPE tissue specimens at a thickness of 6 μm were used to extract DNA. Primers targeting microsatellite markers BAT-25 and BAT-26 and dinucleotide repeats D5S346, D2S123, and D17S250 were used to amplify mononucleotide repeats. Tumors were scored as follows: (1) MSI-high if two or more markers of instability were present, (2) MSI-low if one marker of instability was present, and (3) microsatellite stable (MSS) if none of the markers for MSI were present. For samples with no matched normal tissue, IHC was used to determine mismatch repair status. FFPE tissue specimens were processed using the streptavidin-biotin method with a DAKO LSAB kit (DAKO, Carpinteria, CA). Tumors that yielded negative staining results for at least one of the mismatch repair proteins (MLH1, MSH2, MSH6, or PMS2) were classified as MSI, whereas all others were classified as MSS tumors.

### p53 IHC

p53 immunostaining was performed on FFPE tumor samples using an anti-p53 antibody (1:1500, Dako, Glostrup, Denmark) with a BenchMark XT automatic immunostaining device (Ventana Medical Systems, AZ, USA) and an OptiView DAB IHC Detection Kit (Ventana Medical Systems) according to the manufacturer’s instructions. The percentage of p53 expression in the nucleus was calculated.

### Statistical methods

All statistical calculations were performed in R (v3.5.1, R Foundation for Statistical Computing, Vienna, Austria).

## Results

### Patient characteristics

The characteristics of the 47 patients are summarized in Table 1. The median age was 36 years (range, 22–41), and 25 (53%) patients were male. Of 47 tumors, 38 (81%) were at stage 3 or 4 at diagnosis, and 36 (77%) were located in the left colon. The cases were grouped into mutant *POLE*, MSI, and MSS subtypes. Mutant *POLE* and MSI tumors were more prevalent in male patients and in the left colon. Approximately two-thirds (64%) of the patients had a family history of cancer, and 12 (26%) had a family history of colon cancer (Supplementary Table 1).

**Table 1:** AMC_WES patient characteristics.

Characteristic	Total (*N* = 47)	*POLE* (*N* = 4)	MSI (*N* = 6)	MSS (*N* = 37)
Median age (range)	36 (22–41)	33.5 (24–39)	27 (22–38)	37 (25–41)
*Sex*				
Male	25 (53%)	3	4	18
Female	22 (47%)	1	2	19
*Stage at diagnosis*				
I	1 (2%)	0	0	1
II	8 (17%)	2	2	4
III	24 (51%)	2	3	19
IV	14 (30%)	0	1	13
*Tumor location*				
Right colon	10 (21%)	2	3	5
Left colon	36 (77%)	2	3	31
Multiple	1 (2%)	0	0	1
*MSI status*				
MSI	6 (13%)	0	6	0
MSS	41 (87%)	4	0	37
*Family history of cancer*				
1st degree	9 (19%)	0	1	8
2nd degree	10 (21%)	2	2	6
3rd degree	9 (19%)	0	2	7
4th degree	2 (4%)	0	0	2
No history	17 (36%)	2	1	14
*Family history of colon cancer*				
1st degree	3 (6%)	0	1	2
2nd degree	2 (4%)	0	0	2
3rd degree	6 (13%)	0	4	2
4th degree	1 (2%)	0	0	1
No history	35 (74%)	4	1	30

*MSI* microsatellite instability, *MSS* microsatellite stable.

### Driver mutations in hypermutated samples

**Fig. 1:**
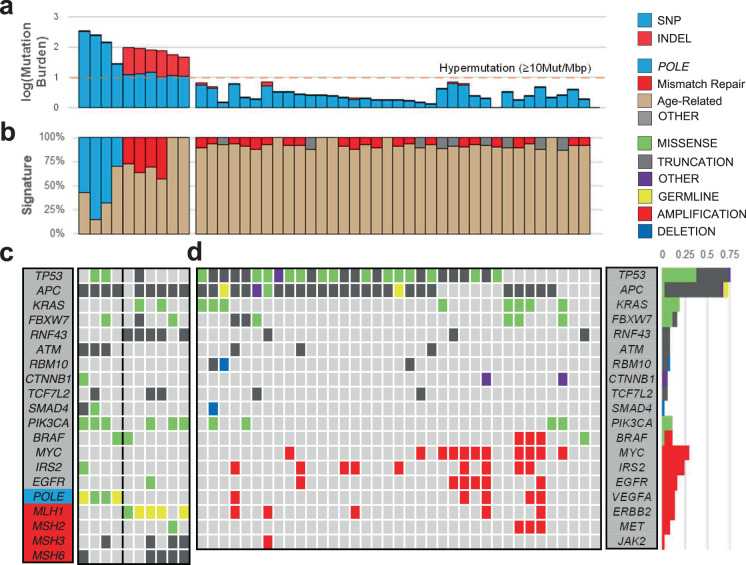
Mutational landscape of early-onset colorectal cancer. **a** Mutation burden of exome samples. Hypermutation was defined as samples with ≥10 mutations per mega base pair. A high indel ratio is notable in samples with mismatch repair gene mutations. **b** Mutation signature ratios of age-related Signature 1, *POLE*-related Signature 10, and mismatch repair-related Signatures 6, 15, 26 combined. **c** Commonly mutated genes in hypermutated samples. **d** Commonly mutated genes in non-hypermutated samples. mutation frequencies of non-hypermutated samples are shown on the right.

As hypermutated CRCs display distinct characteristics, we classified the samples according to their mutation burden as hypermutated (≥10 mutations/Mbp) and non-hypermutated (Fig. 1a). Four hypermutated samples had high proportions of signatures driven by error-prone polymerases (Fig. 1b), and *POLE* mutations were found in all four tumors. Other samples with hypermutation had much higher indel-to-SNP ratios than non-hypermutated samples. These cancers carried mutations in mismatch repair-associated genes (Fig. 1c). Defective mismatch repair was confirmed in all cancer tissues by IHC staining.

Among 53 patients with normal WES data, two harbored germline *APC*-truncating variants, and endoscopy revealed numerous polyps concurrent with germline variants in these patients; the two patients were diagnosed with familial adenomatous polyposis. Among six patients with MSI tumors, three had pathogenic germline variants in *MLH1*, which met the diagnostic criteria for Lynch syndrome. Another patient with MSI cancer carried germline rs876659002, which causes a missense mutation in *MLH1*; this patient had negative IHC results for *MLH1*, and this variant was found to be biallelic in cancer with no other mutations in *MLH1*. Two patients carried germline variants in *POLE*, indicating proofreading polymerase-associated polyposis. Overall, six of ten hypermutated samples carried possible pathogenic germline variants (Supplementary Table 1).

### Common mutations in non-hypermutated samples

**Fig. 2:**
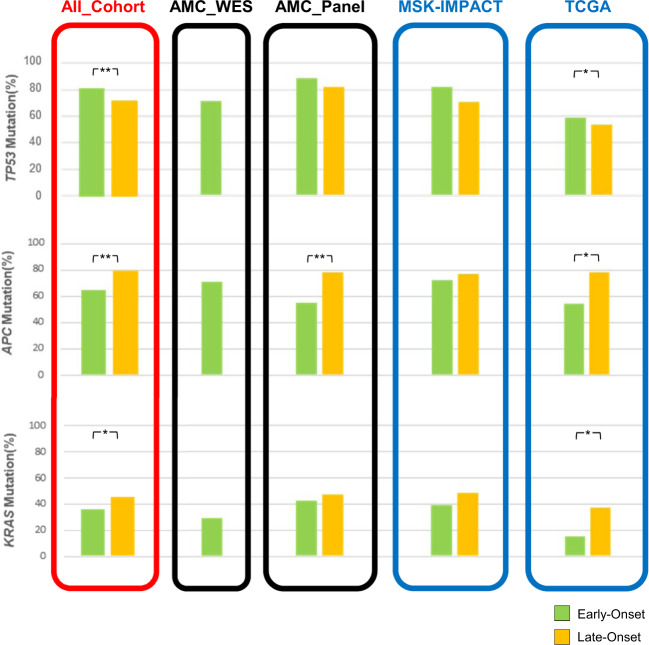
Mutation frequencies between age groups for highly mutated genes. Mutation frequencies of commonly mutated genes. The mutation frequency of the cohorts as a whole is shown on the left. Fisher’s exact *P < 0.05, **P < 0.01.

After excluding two samples diagnosed with familial adenomatous polyposis, we analyzed recurrently mutated genes in 35 non-hypermutated, noncancer syndrome samples (this cohort is hereafter called AMC_WES) (Fig. 1d, Supplementary Table 2). Genes mutated in LO-CRC were also commonly mutated in EO-CRC, albeit with slight differences in the order of mutation frequency. *TP53* was the most commonly mutated gene, and the mutation frequency was higher than that previously reported^27,28^. All mutations suspected to have pathogenicity are listed in Supplementary Table 3. We additionally summarized 833 panel-seq data (AMC_panel) composed of 99 EO-CRC and 734 LO-CRC samples with TCGA and MSK data. The patient characteristics of all cohorts are summarized in Supplementary Table 4. EO-CRC showed a higher frequency of *TP53* mutation (80% vs 72%, Fisher’s exact *P* = 0.03) and lower frequencies of *APC* (65% vs 78%, *P* < 0.001) and *KRAS* (37% vs 45%, *P* = 0.01) mutation than LO-CRC (Fig. 2).

### Integration of mutation and copy number profile

To further characterize the samples, we performed clustering of arm-level copy number changes using WES data. Combined with the mutation data, we found three major groups (Supplementary Table 5): a WGD group, genome-stable (GS) group, and hypermutated group. The WGD group had a higher copy number among all chromosomes. The WGD group also had higher cancer stages than the GS group, though this was not statistically significant (Mann–Whitney *P* = 0.086). The GS group had a significantly lower frequency of copy number gains. The hypermutated group showed minimal copy number-changing events, with no arm-level events.

Focal oncogenic amplification events were also different among the groups (Supplementary Table 6). Focal amplifications of seven or more copies were detected in *MYC* (22%), *IRS2* (17%), and *EGFR* (14%) in the WGD group. However, focal amplification events were not common in the GS and hypermutated groups; these two groups mostly carried point mutations in hotspot regions of driver genes such as *KRAS*, *BRAF*, and *PIK3CA* (Supplementary Table 6).

### Differences between age groups

**Fig. 3:**
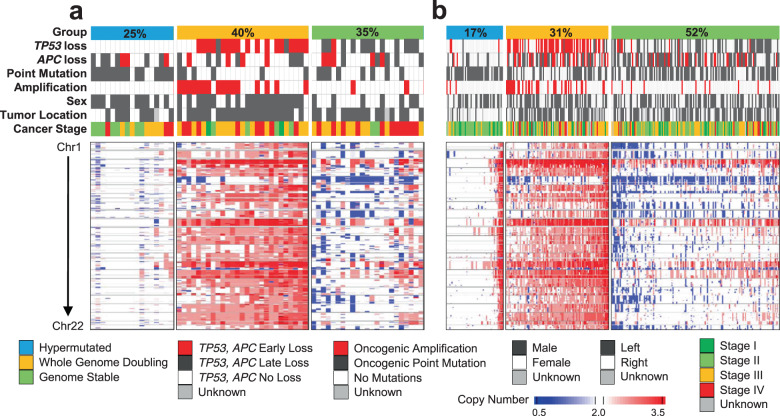
Differences according to age using an integrated data set. Summary of the integrated dataset in **a** early-onset colorectal cancer and **b** late-onset colorectal cancer. A copy number heatmap is presented.

Analysis between age groups in the integrated data sets (Fig. 3) revealed differences in the group ratio. EO-CRC involved a higher proportion of hypermutated samples, presumably owing to the inclusion of patients with predisposing germline variants. For non-hypermutated samples, EO-CRC had a higher rate of WGD than LO-CRC (54% vs 38%, Fisher’s exact *P* = 0.0399), which is in line with our aforementioned data of a higher frequency of *TP53* mutations and lower frequencies of *APC* and *KRAS* mutations in EO-CRC.

Regarding clinical features, EO-CRC occurred more frequently on the left side of the colon than LO-CRC. This tendency was also found when dividing the group into hypermutated and non-hypermutated groups. Subgroup analysis revealed that in women with EO-CRC, the tumor occurred more often on the left side than in women with LO-CRC. Considering the differences between the cohorts, we randomly selected samples to account for stage differences between cohorts, and the aforementioned tendencies remained significant in the stage-matched cohort.

### Relationship between TP53 and WGD

**Fig. 4:**
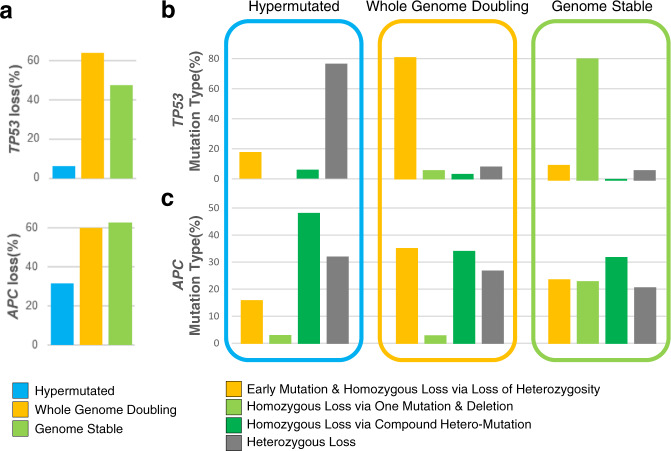
Mutation types in tumor-suppressor genes. **a** Percentages of homozygous loss in *TP53* and *APC* identified from sequencing data. Mutation types in **b** *TP53* and **c** *APC*.

The majority of samples in the WGD group exhibited biallelic loss of *TP53* via copy-neutral LoH, regardless of age. Mutation timing and the degree of functional loss in *TP53* and *APC* were calculated using the variant allele frequency and estimated allele counts. Homozygous loss of *TP53* was detected in 64% of the WGD group, and 81% of *TP53* mutations occurred prior to genome doubling (Fig. 4a, b, Supplementary Table 7). This result is in agreement with previous findings that *TP53* is not only associated with WGD but may also be a prerequisite for genome doubling^33^. On the other hand, only 47% of GS samples showed *TP53* loss, and 81% of the *TP53* mutations had homozygous loss via one mutation and one deletion. The hypermutated group displayed a very low fraction of *TP53* loss.

To obtain additional evidence regarding the timing of *TP53* mutation, we performed IHC for p53 in 26 of the 35 non-hypermutated AMC_WES samples (Supplementary Table 8). The staining patterns of p53 IHC can be divided into three categories: heterogeneous, diffuse strong, and complete-absent^34^. In cases of early *TP53* mutation, p53 IHC should show identical patterns across all tumor cells. Complete absence had high concordance with homozygous loss of *TP53* by truncating mutation, regardless of whether the second hit occurred via LoH or deletion. Except for one sample, all completely absent IHC samples harbored *TP53*-truncating mutations, suggesting a high possibility that the mutation was clonal and occurred early. Samples with no mutations and with missense mutations generally showed heterogeneously and diffuse strong staining patterns, respectively, but the timing could not be assessed based on IHC results.

*APC* is mutated in the early stages of traditional colorectal carcinogenesis. However, early homozygous loss of *APC* was apparent in only one-third of all samples with WGD (Fig. 4a). The ratio and type of functional loss were strikingly different between *TP53* and *APC* (Fig. 4c). Compound heterozygous mutation—two or more different mutations in single cancer—was quite common, accounting for 34%, 32%, and 48% of samples in the WGD, GS, and hypermutated groups, respectively.

### Cause of TP53 mutation

To identify the cause of *TP53* mutation, we used the mutation probability provided from the PHANTM database^26^. Although only single-base substitutions are predicted in the database, more than half of *TP53* mutations (61%, 85/140) in the AMC_WES and TCGA cohorts had a high probability (≥90%) of Signature 1 as the cause of the mutational process. No significant difference in distribution was found in subgroup analyses according to age, subtype, or sidedness.

## Discussion

By integrating mutation, chromosomal copy number, and clinical information, we divided CRC by the carcinogenesis step into three subgroups: (1) a hypermutated group, which includes EO-CRC with predisposing germline variants; (2) a WGD group with early *TP53* mutation, genome doubling, and focal oncogene amplification leading to malignancy; and (3) a GS group, which follows conventional colorectal carcinogenesis, exhibiting point mutations in *APC*, *KRAS*, *TP53*, and deletion of tumor-suppressor genes.

Strong carcinogenic potency is essential for a tumor to grow large enough and to be diagnosed in the early years of life. Therefore, EO-CRC primarily consists of cancers with high tumorigenic capabilities. One mechanism of early-onset is carrying cancer-predisposing germline variants. As hereditary cancer syndromes only need one additional mutation for cancer initiation, cancer syndrome patients thus have a higher chance of developing cancer and develop it relatively earlier.

As *TP53* is the gatekeeper of damaged cells, the early loss of *TP53* in the WGD group is another way to gain a greater level of survival fitness within a short time period. Recent publications have reported the relationship between *TP53* and aneuploidy. *TP53* is significantly associated with WGD, and *TP53* mutation occurs earlier than WGD in 97.3% of associated cases^33^. The rate of WGD in colon cancer is high among multiple cancer types. One group calculated aneuploidy with TCGA data and found *TP53* to be the sole gene correlating with aneuploidy^35^. The mechanism of *TP53* mutation, leading to WGD appears to be associated with increased tolerance to missegregation events^36,37^. *TP53* activation during aneuploidy events is not clear, but the loss of function in *TP53* is expected to reduce cell death after segregation errors. In precancerous lesions, *TP53* mutation is more frequently found than *TP53* copy-neutral LoH^38^, which can also be a clue of the temporal order of *TP53* mutation and WGD. In the normal epithelium, WGD is rarely found compared with *TP53* mutation or *TP53* LoH, suggesting that *TP53* mutation and LoH occur earlier than WGD^39^.

The GS subgroup is similar to conventional CRC. Copy number events in the GS subgroup are mostly deletions, and focal amplifications are rare. The reason for the early onset of this tumor type is unknown. A possible explanation may simply be random mutations occurring at critical sites.

Li-Fraumeni syndrome (LFS), a genetic disorder involving germline variants of *TP53*, entails malignancies such as osteosarcoma, breast cancer, brain cancer, and adrenal gland cancers. Colon cancer is relatively infrequent in patients with LFS, occurring in ~2.8% of cases^40^. One CRC report involves an LFS patient who also had numerous precancerous lesions and adenocarcinomas^41^. As thorough genomic profiling of LFS-induced CRC has yet to be carried out, we could not find any data regarding chromosomal state; nevertheless, *TP53* mutation causes early onset of CRC^42^, and *TP53* has high carcinogenic potency compared with other CRC-associated genes.

Colitis-associated colorectal cancer (CAC) is caused by inflammatory bowel disease (IBD) and is associated with the severity and duration of IBD^43^. The molecular aspect of CAC is similar to that of the WGD group: high *TP53*, low *APC*, and low *KRAS* mutation frequencies^44,45^. Mutation in *TP53* is a typical early event in CAC, as a mutation of this gene is found not only in carcinoma tissues but also in tissues showing dysplasia or even in an inflamed but nondysplastic epithelium^43^. Chromosomal instability and WGD are also common in CAC, and studies have shown high levels of chromosomal aneuploidy as well as small regions with high levels of focal amplification in CAC^46,47^. Although our EO-CRC cohort did not include patients with IBD, the carcinogenesis of CAC is strikingly similar to that of sporadic CRC in the WGD group and may provide additional clues to the development of early malignancies.

A polypoid adenoma is a conventional precursor to CRC and is commonly is associated with *APC* mutations, whereas nonpolypoid flat lesions classified as the lateral spreading tumor nongranular type (LST-NG) display different molecular traits. Several studies have compared the molecular characteristics of these precancerous lesions^48–50^, showing that mutations in *KRAS* and *APC* are more common in polyploids than in LST-NG. Despite interstudy discrepancies with regard to the mutation rates of *TP53*, LST-NG tends to have more *TP53* mutations than other lesions. Indeed, one study reported that *TP53* mutation was present in 74% of LST-NG samples with submucosal invasion and in 40% of LST-NG intraepithelial cancer samples, suggesting that *TP53* mutation occurs in earlier stages of tumorigenesis^51^. The aforementioned case of a patient with LFS and CRC also involved >10 LST-NG lesions throughout the colorectum.

The reason for the driver mutations occurring early in age is unclear, but most *TP53* mutations were predicted to be induced through mutational signature 1. Signature 1 is produced by spontaneous deamination, and the number of mutations correlates with age and cell division rate. If *TP53* mutation occurs from natural replication error during cell division and assuming that the replication error rate is similar across individuals, the mutational process alone cannot explain the rapid increase in the incidence of EO-CRC. We speculate that an extrinsic factor may cause the acceleration of cell division, which in turn increases the chance of replication error. As prolonged inflammation leads to *TP53* mutation in patients with IBD, the extrinsic factor may trigger localized inflammation and induce carcinogenic effects. By sequencing the normal colon at the single-crypt level^38^, researchers have found patches of mutations sharing common signatures along the colon. This unknown signature was quite common and presumed to occur before the age of 10 by extrinsic mutagen. Thus, the first oncogenic mutations may occur at an early age and develop slowly, a pattern similar to those of cancers arising from predisposing germline variants.

Therapeutic suggestions may be made based on EO-CRC subgroups. Patients with hypermutated tumors may be considered candidates for immune checkpoint inhibitors, which are effective in patients with MSI CRC. For patients in the WGD group, novel treatments based on driver gene amplification can be considered using drug repositioning and development. The anti-HER2 treatment has also been shown to be effective in CRCs with *ERBB2* amplification^52^. In addition, *MET* amplification has been suggested to be associated with poor prognosis and acquired resistance to anti-*EGFR* treatment. The antitumor effect of several anti-*MET* agents with proven efficacy in other cancers may be assessed in CRCs with *MET* amplification^53^. Targeting *MYC* has been suggested as a therapeutic strategy in several cancers, and many preclinical studies have demonstrated the potential anticancer effect of *MYC* inhibition^54^. Moreover, alternative approaches to MYC blockade are under exploration to overcome the so-called “undruggable” protein structure of MYC. A standard treatment strategy may be applied for patients in the GS group, who have molecular features similar to those with LO-CRC.

Although the incidence of EO-CRC is increasing worldwide, our findings have limited generalizability due to the small cohort size and narrow ethnicity studied. Further research is crucial to identify the common aspects of EO-CRC among patients of different ethnic backgrounds. The descriptive nature of our study is another limitation, as we could only speculate on causality. We encourage researchers to study other aspects of EO-CRC, such as lifestyle, food, and drug intake, as well as cancer-related socioeconomic factors. Large-scale studies involving multiple aspects should ultimately be conducted to delineate the cause of early-onset cancer.

## Supplementary information

Supplementary Table

## Data Availability

Exome-sequencing data are available at Sequence Read Archive under number PRJNA602165. Panel data are not publicly available because panel sequencing was performed for individual patient treatment decisions. We used deidentified sequencing data for this analysis based on IRB’s decision; the deidentified data are available from the corresponding author on reasonable request.

## References

[1] Siegel, R. L. et al. Colorectal cancer incidence patterns in the United States, 1974–2013. *J. Natl. Cancer Inst*. **109**, djw322 (2017). 10.1093/jnci/djw322 PMC6059239 28376186

[2] Siegel RL (2019). Global patterns and trends in colorectal cancer incidence in young adults. Gut.

[3] Sung JJY (2019). Increasing trend in young-onset colorectal cancer in Asia: more cancers in men and more rectal cancers. Am. J. Gastroenterol..

[4] Wolf AMD (2018). Colorectal cancer screening for average-risk adults: 2018 guideline update from the American Cancer Society. CA Cancer J. Clin..

[5] Murphy CC, Lund JL, Sandler RS (2017). Young-onset colorectal cancer: earlier diagnoses or increasing disease burden?. Gastroenterology.

[6] Goel A (2010). Low frequency of Lynch syndrome among young patients with non-familial colorectal cancer. Clin. Gastroenterol. Hepatol..

[7] Pearlman R (2017). Prevalence and spectrum of germline cancer susceptibility gene mutations among patients with early-onset colorectal cancer. JAMA Oncol..

[8] Lieu CH (2019). Comprehensive genomic landscapes in early and later onset colorectal cancer. Clin. Cancer Res..

[9] Serebriiskii IG (2019). Comprehensive characterization of RAS mutations in colon and rectal cancers in old and young patients. Nat. Commun..

[10] Bolger AM, Lohse M, Usadel B (2014). Trimmomatic: a flexible trimmer for Illumina sequence data. Bioinformatics.

[11] Li H, Durbin R (2009). Fast and accurate short read alignment with Burrows-Wheeler transform. Bioinformatics.

[12] McKenna A (2010). The Genome Analysis Toolkit: a MapReduce framework for analyzing next-generation DNA sequencing data. Genome Res..

[13] Talevich E, Shain AH, Botton T, Bastian BC (2016). CNVkit: genome-wide copy number detection and visualization from targeted DNA sequencing. PLoS Comput. Biol..

[14] Layer RM, Chiang C, Quinlan AR, Hall IM (2014). LUMPY: a probabilistic framework for structural variant discovery. Genome Biol..

[15] Chiang C (2015). SpeedSeq: ultra-fast personal genome analysis and interpretation. Nat. Methods.

[16] Ye K, Schulz MH, Long Q, Apweiler R, Ning Z (2009). Pindel: a pattern growth approach to detect break points of large deletions and medium sized insertions from paired-end short reads. Bioinformatics.

[17] Wang K, Li M, Hakonarson H (2010). ANNOVAR: functional annotation of genetic variants from high-throughput sequencing data. Nucleic Acids Res..

[18] Ramos AH (2015). Oncotator: cancer variant annotation tool. Hum. Mutat..

[19] Cingolani P (2012). A program for annotating and predicting the effects of single nucleotide polymorphisms, SnpEff: SNPs in the genome of Drosophila melanogaster strain w1118; iso-2; iso-3. Fly (Austin).

[20] Rosenthal R, McGranahan N, Herrero J, Taylor BS, Swanton C (2016). DeconstructSigs: delineating mutational processes in single tumors distinguishes DNA repair deficiencies and patterns of carcinoma evolution. Genome Biol..

[21] Cleary JG (2014). Joint variant and de novo mutation identification on pedigrees from high-throughput sequencing data. J. Comput. Biol..

[22] Purcell S (2007). PLINK: a tool set for whole-genome association and population-based linkage analyses. Am. J. Hum. Genet..

[23] Riester M (2016). PureCN: copy number calling and SNV classification using targeted short read sequencing. Source Code Biol. Med..

[24] Landrum MJ (2018). ClinVar: improving access to variant interpretations and supporting evidence. Nucleic Acids Res..

[25] Li Q, Wang K (2017). InterVar: clinical interpretation of genetic variants by the 2015 ACMG-AMP guidelines. Am. J. Hum. Genet..

[26] Giacomelli AO (2018). Mutational processes shape the landscape of TP53 mutations in human cancer. Nat. Genet..

[27] Hoadley KA (2018). Cell-of-origin patterns dominate the molecular classification of 10,000 tumors from 33 types of cancer. Cell.

[28] Yaeger R (2018). Clinical sequencing defines the genomic landscape of metastatic colorectal cancer. Cancer Cell.

[29] Chun SM (2018). Next-generation sequencing using s1 nuclease for poor-quality formalin-fixed, paraffin-embedded tumor specimens. J. Mol. Diagn..

[30] Kim JE (2019). Mutation burden and index for detection of microsatellite instability in colorectal cancer by targeted next-generation sequencing. J. Mol. Diagn..

[31] Fernandez NF (2017). Clustergrammer, a web-based heatmap visualization and analysis tool for high-dimensional biological data. Sci. Data.

[32] Carter SL (2012). Absolute quantification of somatic DNA alterations in human cancer. Nat. Biotechnol..

[33] Bielski CM (2018). Genome doubling shapes the evolution and prognosis of advanced cancers. Nat. Genet..

[34] Kobel M (2019). Interpretation of P53 Immunohistochemistry in endometrial carcinomas: toward increased reproducibility. Int. J. Gynecol. Pathol..

[35] Taylor AM (2018). Genomic and functional approaches to understanding cancer aneuploidy. Cancer Cell.

[36] Dewhurst SM (2014). Tolerance of whole-genome doubling propagates chromosomal instability and accelerates cancer genome evolution. Cancer Discov..

[37] Soto M (2017). p53 prohibits propagation of chromosome segregation errors that produce structural aneuploidies. Cell Rep..

[38] Lee-Six H (2019). The landscape of somatic mutation in normal colorectal epithelial cells. Nature.

[39] Martincorena I (2018). Somatic mutant clones colonize the human esophagus with age. Science.

[40] Wong P (2006). Prevalence of early onset colorectal cancer in 397 patients with classic Li-Fraumeni syndrome. Gastroenterology.

[41] Yoshida T (2017). The features of colorectal tumors in a patient with Li-fraumeni syndrome. Intern. Med..

[42] Yurgelun MB (2015). Germline TP53 mutations in patients with early-onset colorectal cancer in the colon cancer family registry. JAMA Oncol..

[43] Xie J, Itzkowitz SH (2008). Cancer in inflammatory bowel disease. World J. Gastroenterol..

[44] Robles AI (2016). Whole-exome sequencing analyses of inflammatory bowel disease-associated colorectal cancers. Gastroenterology.

[45] Yaeger R (2016). Genomic alterations observed in colitis-associated cancers are distinct from those found in sporadic colorectal cancers and vary by type of inflammatory bowel disease. Gastroenterology.

[46] Habermann JK (2003). Pronounced chromosomal instability and multiple gene amplifications characterize ulcerative colitis-associated colorectal carcinomas. Cancer Genet. Cytogenet..

[47] Baker AM (2019). Evolutionary history of human colitis-associated colorectal cancer. Gut.

[48] Konda K (2014). Distinct molecular features of different macroscopic subtypes of colorectal neoplasms. PLoS ONE.

[49] Sakai E (2014). Methylation epigenotypes and genetic features in colorectal laterally spreading tumors. Int. J. Cancer.

[50] Sugai T (2017). Analysis of molecular alterations in laterally spreading tumors of the colorectum. J. Gastroenterol..

[51] Sakai E (2016). TP53 mutation at early stage of colorectal cancer progression from two types of laterally spreading tumors. Cancer Sci..

[52] Sartore-Bianchi A (2016). Dual-targeted therapy with trastuzumab and lapatinib in treatment-refractory, KRAS codon 12/13 wild-type, HER2-positive metastatic colorectal cancer (HERACLES): a proof-of-concept, multicentre, open-label, phase 2 trial. Lancet Oncol..

[53] Safaie Qamsari E (2017). The c-Met receptor: implication for targeted therapies in colorectal cancer. Tumour Biol..

[54] Chen H, Liu H, Qing G (2018). Targeting oncogenic Myc as a strategy for cancer treatment. Signal Transduct Target Ther..

